# The making of the *Homo Polaris*: human acclimatization to the Arctic environment and Soviet ideologies in Northern Medical Institutions

**DOI:** 10.1080/2201473X.2023.2274673

**Published:** 2023-10-30

**Authors:** Dmitry V. Arzyutov

**Affiliations:** Department of Slavic and East European Languages and Cultures, The Ohio State University, Columbus, OH, USA

**Keywords:** Human acclimatization, Industrialization, Soviet medicine, indigenous peoples, settler colonialism, Cold War, Soviet North, Siberia

## Abstract

This paper examines the history of the Soviet human acclimatization project in the North and Siberia, which spanned from medical experiments in Stalin’s forced labor camps to the subsequent wave of industrialization in the region. The author argues that human acclimatization in the North was a settler colonial science project aimed at facilitating Russian administrators and engineers in asserting control over the territory and its resources, while creating a new homogeneous ‘indigenous population’ in Siberia and the North. This envisioned population, referred to as *Homo Polaris* by the author, was intended to emerge through a two-way transformation: the adaptation of Indigenous peoples into Soviet ideologies and practices, and the acclimatization of settlers coming from the European part of the country to the Arctic environment. Although the administrators and medical doctors were unable to achieve this biopolitical objective, the complexities and dialogues surrounding these transformations shed light on the late Soviet settler-colonial ideologies and their impact on social life in Siberia from the 1950s to the 1980s. The research is based on a comprehensive analysis of both published and archival works by scholars involved in the project.

The industrial development of the North and Siberia during the Cold War was one of the most dramatic episodes in late Soviet history, both in terms of its epic scale and its environmental and social consequences. This key planetary region has undergone a transformation beyond recognition. Some environmental historians argue that these changes have contributed to what is now known as the Anthropocene, an era characterized by global environmental and geological disruption caused by human technological activities over the past few decades.^1^

The Siberian industrial development inherited the material and social infrastructure of Stalin’s forced labor camps (GULag^2^) and partly what left behind after the early Soviet colonization of the North.^3^ It also attracted a massive migration of people from the Western and Southern regions of the Soviet Union to Siberia and the North, which significantly altered the demographic profile of the region.^4^ However, what is less known is that this new wave of industrialization was accompanied by a large-scale medical project. It brought together metropolitan scholars and local physicians to assist newcomers with their acclimatization.^5^

The present paper explores the intellectual history of the project that partially originated in the early years of Stalin-era development of the North.^6^ It was supplemented by medical research conducted in the GULag camps during the 1930s and further advanced during the new wave of massive industrialization in the 1970s and 1980s. The article primarily focuses on the latter period of the project, spanning from the mid-1950s to the mid-1980s. This period was crucial in shaping the region’s economic history, as Siberia emerged as a major supplier of gas to the West.^7^ Geographically, the research focuses on the territory from Arkhangelsk to the Taymyr Peninsula, with special attention given to Novosibirsk as the late Soviet center for acclimatization studies.

Additionally, the acclimatization project was intricately linked to larger late Soviet modernization initiatives, including Northern urbanization, the sedentarization of nomadic and semi-nomadic Indigenous communities, the development of circumpolar agriculture, and the expansion of nature reserves, among others. Thus, the Siberian acclimatization project was not solely a medical endeavor but was deeply embedded within social and political contexts. Its objective was to adapt the bodies of non-Siberian settlers to the northern environment, which they, in turn, conquered and transformed.^8^ The Indigenous communities, initially overlooked in the history of the acclimatization project, faced immense pressure to ‘modernize’ and ‘Sovietize’ their way of life,^9^ as reflected in the works of Soviet medical doctors.

Although the goal of achieving bilateral human acclimatization to the Northern environment and Soviet ideologies was challenging for various reasons, including political bias toward Indigenous peoples and their knowledge, it dominated discussions surrounding industrial projects in the North and received significant state sponsorship.^10^ At a practical level, scholars assumed that settlers from the European part of the country^11^ would need to acquire knowledge and even physical characteristics from the ‘backward’ Indigenous population. Despite their exclusion from decision-making in industrialization projects and their officially recognized low position on the social evolution ladder, Indigenous peoples remained examples for doctors of successful survival under harsh northern conditions.^12^ Medical scholars, influenced by Soviet identity politics and its classification of ethnic groups,^13^ consistently documented the ethnic identity of their patients. However, in their publications, they often preferred to group different Indigenous communities under the collective term *korennye* (natives or Indigenous). This bias resulted in little interest in mixed communities or families in Siberia and the North. Consequently, they viewed the acquisition of acclimatization characteristics by settlers as a process facilitated by scholars rather than Indigenous neighbors. These scholars measured bodies, weather, climate, houses, and clothing, and provided advice on how to become ideal settlers in the North.

Medical doctors and administrators envisioned a homogeneous collective body as a ‘new generation of northerners’ (R. *severi͡ane*)^14^ or even the future ‘indigenous population’ (R. *korennoe naselenie*) of the North.^15^ They believed that these individuals should be socially constructed, medically supported, and successfully settled in and around Siberian and Northern industrial areas. In other words, as planned, these people would become the dominant labor force, taking complete control over the region and extracting its mineral resources for the benefit of the Soviet country. In this paper, I coin the term *Homo Polaris* to encompass the various terminologies used in Soviet publications regarding the process of human acclimatization.

As demonstrated in this paper, the encounters and dialogues surrounding *Homo Polaris* reveal settler-colonial ideologies that have often been overlooked in late Soviet Siberian studies.^16^ On one hand, these ideologies were rooted in the long and broad history of colonial medicine^17^ and, at a national level, of Russian settler colonialism in Siberia.^18^ Despite criticism of these ideologies and practices in the 1920s,^19^ they resurfaced in public and academic discourse as a Russian nationalist ideology, particularly vocalized since the late 1930s.^20^ On the other hand, these ideologies were tempered by the officially proclaimed early Soviet affirmative action policies,^21^ the echoes of which reverberated throughout the late Soviet Union^22^ due to the politics of ‘revival’ (R. *vozrozhdenie*) of ethnic and Indigenous cultures across the country.^23^ The emerging hybridity of these state-sponsored attitudes turned late Soviet settler colonialism into a space for ongoing discussions, including those concerning post-Soviet discrimination against ethnic and Indigenous groups in modern Russia and its recently annexed territories.^24^

Lorenzo Veracini, a historian of settler colonialism, states that ‘settler colonialism is about turning a place and a specific human material into something else, and, paradoxically and simultaneously, about a specific human material that remains true to itself in a place that is “other”^25^’. Travis Hay, a historian of settler colonial medicine in Canada, supplements Veracini’s observation with a bottom-up perspective, formulating it as follows:

[t]he science of settler colonialism is a three-step process: first, Indigenous peoples are subjected to the relocating, malnourishing, and traumatizing impacts of federal Indian policy; second, settlers are sent on research trips to observe and gather data on the impacts of this intervention; and third, these data are interpreted and assembled in ways that blame Indigenous peoples for their own poor health outcomes.^26^

Despite some distinctions found in early Soviet politics, such as positive discrimination toward ethnic groups and the carceral genealogy of medical research in the 1930s, the Siberian acclimatization project followed similar steps. These steps included Russian colonial dominance over the territory and peoples, as well as the criticism and study of healthcare practices of Siberian natives that were not yet ‘civilianized’. Interestingly, this latter aspect can be found in the Imperial and early Soviet ethnographic and medical literature about Siberian settlers. This category included both those who arrived in the late nineteenth and early twentieth centuries (R. *pereselentsy*)^27^, as well as those who came centuries before that, known as ‘old settlers’ or *starozhily* or *chaldony* in Russian.^28^

During the late Soviet period, the Siberian human acclimatization project became entangled with a diverse range of ideas and concepts. These encompassed Ivan Pavlov’s psychophysiological typology, as well as the notions of the biosphere and noosphere borrowed from the works of Russian geochemist Vladimir I. Vernadskiy. Despite their ambitious and speculative nature, and their incorporation into medical academic literature by the 1980s, these frameworks tended to overshadow Indigenous knowledge, despite its crucial role in the development of Arctic and Siberian medical expertise. Consequently, my archival research encountered some difficulties in identifying local and Indigenous perspectives within materials related to the acclimatization projects. However, a careful examination of the writings of Soviet acclimatization scholars enabled me to discern instances where they openly or subtly referenced the practices and knowledge of Indigenous groups in Siberia and the North. Specifically, they either sought to adapt these practices for their settler patients or, conversely, dismissed them as culturally inappropriate. Through these often concise and not well-articulated passages, my objective is to shed light on the Indigenous knowledge and practices that were not given individual recognition but ultimately influenced settler colonial ideas regarding medical projects. Moreover, in some parts of the present articles, I align those passages with Indigenous and local anthropology and history to reveal the silenced voices.

To understand the political and epistemic transformations that occurred in late Soviet North and Siberia, affecting both Indigenous communities and settlers from the European part of the country, I employ the term ‘making’, which is also reflected in the article’s title. By using this term, I refer to the socially active process^29^ involving Soviet administrators, medical doctors, and both Indigenous and ‘Russian’ settler communities. While their voices were never equal, as I indicated above, the configurations of their dialogues and numerous actions and conversations during numerous conferences, discussions, and field research merely outlined the path forward and did not necessarily guarantee results, such as imagined exerting control over the bodies of those living in the Soviet North. Therefore, the present article primarily focuses on the entangled genealogies of the project.

To provide context for the extensive acclimatization program in the 1970s, it is necessary to examine preceding episodes.

## I.

The famous 20th Congress of the Communist Party of the Soviet Union (14–25 February 1956),^30^ where Nikita Khrushchev secretly denounced the ‘cult of personality’ surrounding Iosif Stalin, was also notorious for its public statement regarding the mobilization of natural resources of the eastern regions of the country. In his speech, Nikita Khrushchev stated,

Here, comrades, how profitable it is for us to develop the energy resources of the east [of the country. – D.A.] more widely! In the next ten years, we must turn Siberia into the largest base of the Soviet Union for coal mining and electricity production, the main base for heat-intensive and energy-intensive industries, especially the production of aluminum, magnesium, and titanium, as well as electrometallurgy, coal chemistry and electrochemistry. (Applause)^31^

That applause was followed by the new eastern turn of Soviet industrial politics, which entailed a renewed assault on the landscapes and local communities of the North and Siberia. Soviet ministries and the Academy of Sciences began developing new research plans for Siberia, which included the construction of new mines and hydropower plants. As I demonstrate in this section of my article, alongside technological advancements, scholars, and administrators also emphasized the importance of the ‘human force’ and scientifically supported medical care. This was conceptualized through the notion of human acclimatization, which among other Soviet state initiatives, allows us today to uncover the late Soviet settler-colonial ideologies in the North and Siberia.

That project relied on the infrastructure inherited from the GULag, a large network of forced labor camps across the Soviet Union during the Stalin regime, as well as other projects left behind the early Soviet wave of colonization in the region including the relocated industrial plants and factories from Ukraine and other European regions to Siberia and the North during the Second World War.^32^ That infrastructure also encompassed former carceral medical institutions and people working there.^33^ Many of those medical scholars, who would write about Arctic acclimatization in the early 1950s, went through the GULag system themselves. Upon their release, some were even re-employed by the same clinics to continue their research during Khrushchev’s ‘thaw’^34^. One such scholar was physician Grigoriy M. Danishevskiy (1890–1971). He, along with his fellow inmates and some civilian colleagues in *Pechorlag* (The Correctional Labor Camp on the Pechora River in the Komi Republic in the Russian North) initiated research on Arctic acclimatization in their camp clinic.^35^ They conducted vitamin studies to aid prisoners in surviving the harsh conditions of the Arctic labor camps.^36^ Furthermore, Grigoriy Danishevskiy and his team, including Yuriy I. Lakoza, Boris V. Komlev, Nikolai A. Veriga, among others, implemented various agricultural practices near the hospital to provide inmate patients with fresh vegetables.^37^ As noted by Golfo Alexopoulos, a historian of Stalin’s GULag medicine,

Medical researchers [in GULag. – D.A.] were charged with various tasks: to improve diet, nutrition, and disease treatment, to enable colonization of the Soviet Union’s northern territories, and to advance the Stalinist goal of a self-financing prison labor camp system.^38^

The work of Grigoriy Danishevskiy was part of that system, focusing on vitamins and climate. Faced with the scarcity of meat and plant-based food in the northern GULag camps, Grigoriy Danishevskiy had to concentrate on extracting vitamin-rich elements from Arctic plants^39^ and addressing issues related to disadaptational meteoneuroses (R. *dizaptat͡sionnye meteonevrozy*). By disadaptational meteoneuroses, Grigoriy Danishevskiy referred to failed acclimatization resulting from the compromised health conditions of his patients (read: fellow inmates).^40^ Consequently, he emphasized the significance of ‘advancing cultural vitamin-containing flora’ in the North^41^, as it could enhance the living conditions of Arctic dwellers and prevent disadaptational meteoneuroses. This prison genealogy of settler colonial science of human acclimatization placed both groups, those involuntarily confined in Stalin’s labor camps and Indigenous communities who experienced similar hardships due to forced collectivization and Stalin’s persecutions, in comparatively equal conditions on either side of the barbed wire fence.

Furthermore, tuberculosis, scurvy, or dysentery frequently crossed that fence^42^ due to the very low level of state medicine in general and the unavoidable relations across the GULag islands and the ‘outside’ world. Bearing this in mind, Grigoriy Danishevskiy listed the herbs with healing properties against those and other ‘social’ diseases.^43^ No wonder that this list intersected the ethnobotanical knowledge of Komi and other local and Indigenous communities documented by Indigenous and local anthropologists.^44^ Even today, the perception of tuberculosis, for example, still remains closely associated with the carceral milieu.^45^ And hence, both Siberian Indigenous and settler colonial communities share the knowledge and, at times, practice of dog meat (R. *sobachatina*) consumption as a means to combat tuberculosis,^46^ despite its generally perceived inappropriateness in everyday life.^47^ In narratives I heard as a Siberia-born historian and anthropologist during my ethnographic fieldwork among Altaians and Nenets and also among ‘Russian’ settlers, that practice has been always associated with prisons and prisoners.

Thus, despite the initial disregard for the knowledge of Indigenous peoples – particularly Komi and Nenets, whose nomadic camps and settled villages neighbored many of the northern GULag camps in the European part of the country^48^ – early works by Grigoriy Danishevskiy prompted the publication of articles in official Soviet magazines about how local communities were able to survive in the ‘isolated’ Arctic tundra. As a medical doctor for the inmates, he organized lectures and even radio broadcasts on human acclimatization.^49^ Most of those articles focused on improving the nutrition and living conditions of settlers during the Second World War.^50^ That minor medical interest in Indigenous knowledge for human acclimatization persisted after the war.^51^ This does not mean that the post-war settlers who arrived in the North, about whom Soviet scholars discussed as early as 1945^52^ in the context of the first human security programs in the Arctic,^53^ fully adopted the lifestyle of Siberian or northern natives. However, some Indigenous food practices and botanical knowledge were partially incorporated into the early acclimatization programs.^54^ These subtle intersections laid the foundation for the initial conceptualizations of *Homo Polaris*. In a recent chapter, Cassandra Cavanaugh highlights similar discussions that took place in the late 1920s and early 1930s in the field of Central Asian physical anthropology and medicine, where scholars reached similar conclusions. During those debates, they transitioned from the belief that acclimatization of settlers coming from the European part of the country to the Central Asian environment was fundamentally impossible, to integrating elements of Indigenous knowledge into scientific medical research to facilitate the process of human acclimatization.^55^

Upon the death of Iosif Stalin in 1953 and the subsequent liberalization of social and political life in the country, the GULag clinics and medical laboratories and clinics underwent a silent transformation into a network of Northern ‘civic’ clinics.^56^ These clinics emerged in Archangelsk, Murmansk, Norilsk, and other former GULag ‘islands’. Some of these clinics became involved in the initial projects on human acclimatization in the North. Despite the altered focus of those renovated hospitals on human health, unlike what we can see in the GULag medical records,^57^ they both operated with physical labor capability (R. *fizicheskai͡a trudosposobnost’*) as a biopolitical category bridging the forced labor camps with the new wave of industrial development of the North. Moreover, the very procedure of documenting of human physical conditions remained stable despite the social and political changes the region had gone through. Historians of GULag remind us that the details of medical treatment were quite poorly documented and remained obscure for a detailed analysis.^58^ However, what significantly differed is the settler colonial idea of building a ‘new indigenous population’, unlike the colonial attitude in GULag where prisoners either had to die in the frozen land or, if they survived, to return home upon the release.

After his release and full political rehabilitation in 1956, Grigoriy Danishevskiy began working in a similar hospital in Pechora town as part of the mentioned *Pechorlag*. During his time there, he completed and published his major work on human acclimatization in the North *Akklimatizat͡sii͡a cheloveka na Severe* [Human Acclimatization in the North].^59^ This book was soon supplemented by the first translations of English-language military-based works on human adaptation and acclimatization in the non-Soviet sector of the Arctic.^60^ Soviet medical doctors were familiar with some of these works, which they cited in their articles and books.^61^ These developments, along with the first conferences on the subject held in Moscow, Murmansk, Archangelsk, and Norilsk,^62^ shaped Soviet acclimatization scholarship. Intellectually, as a settler-colonial idea, the acclimatization project, since its first academic articulations, intersected various scholarly fields, ranging from Ivan Pavlov’s theory of the second signal system (the function of language in psychopathology) to ideas inherited from social hygiene discussions in the 1920s. To illustrate these intersections, I will provide some quotations from Grigoriy Danishevskiy’s books. In 1955, he wrote,^63^

The most comprehensive stimulus according to I[van] P. Pavlov is a word, the audible and visible word of radio, newspapers, magazines, and books [that are] in the Red Corner (*Krasnyĭ ugolok*)^64^ of a nomadic reindeer dwelling (*chum*^65^). It changes, according to the [Pavlov’s] doctrine of the second signaling system, the reactivity of the cerebral cortex and all the behavior of a reindeer herder nomadizing with a reindeer herd.

Some years later, Grigoriy Danishevskiy further explored the intersections between Soviet progressivist science ideologies and Indigenous knowledge inherited from the past,^66^

The ancestors of the present-day northern [Indigenous] peoples could only minimally utilize the methods of active environmental transformation [R. *vneshni͡ai͡a sreda*] and social adaptation to it, which social life equipped humanity with during its historical development towards modern progress, enlightenment, scientific advancements, technological innovations, and cultural achievements. […] Of course, it would be a mistake to assume that long-standing biological stigmas, genetically ingrained in numerous past generations, will be eradicated as if by magic [R. *po shchuch'emu velenii͡u*; Lit. *upon pike's will*], and that complete physical improvement [R. ozdorovlenie] will suddenly occur among the Indigenous peoples [R. *malye narody*, Lit. small-numbered peoples] of the North. This process will undoubtedly require a significant amount of time. However, there is no doubt that the political, economic, and cultural prerequisites that expedite the population's health improvement are exerting an increasingly influential impact.

This quotation illustrates that in the 1950s, Arctic Indigenous peoples were already considered fully acclimatized to the Northern climate in a biological sense. However, their social and cultural practices were deemed insufficiently Soviet by metropolitan administrators and medical doctors. They aimed to ‘rectify’ Indigenous communities through upcoming changes in their ways of living. This is where settler-colonial ideologies merged the biological and social aspects. Furthermore, the phrase ‘human acclimatization of the North’ in these works did not dictate a single meaning of ‘human’ to Soviet scholars. On the contrary, scholars explicitly discussed two distinct directions of acclimatization. In the discussions and translations of the 1960s, both Soviet scholars and their North American colleagues preferred to differentiate between ‘migrants’ and the ‘Indigenous population’ based on their habits and biological adaptabilities as perceived by the researchers. The first category referred to labor migrants who lacked Arctic experiences, while the second category encompassed Indigenous peoples who were considered experts in Arctic survival but held a lower social evolutionary position compared to ‘Russian’ settlers. While the first category required medical care for physical adaptation to the new environment, the second category required social care to facilitate their integration into the Soviet ‘family of peoples’ on equal terms.^67^

**Figure 1.**
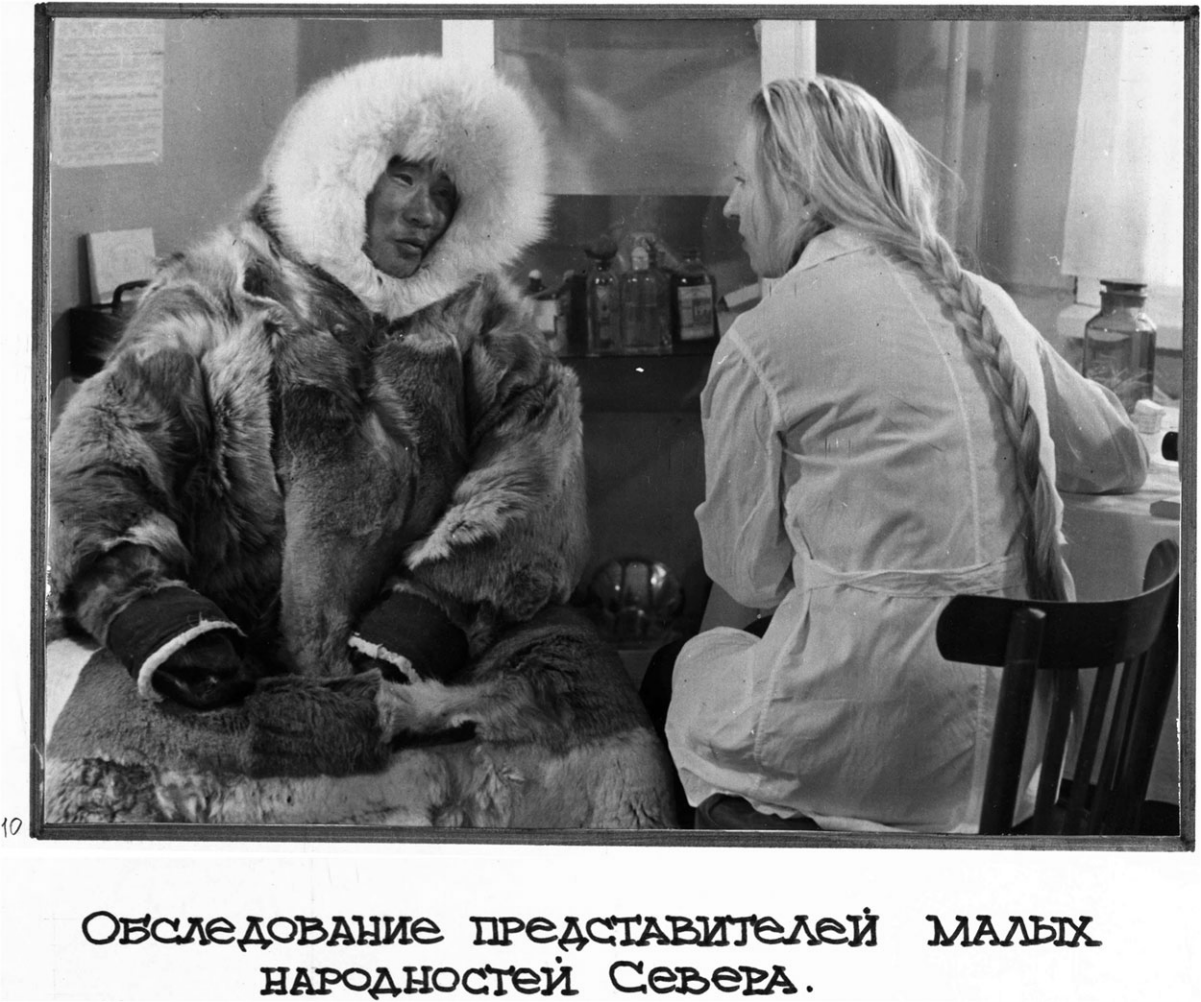
‘The medical research of representatives of small[-numbered] peoples [the Soviet term used for Indigenous peoples. – *D.A*.] of the North’. The 1970–1980s. Taymyr (?). Vlail’ Kaznacheev’s photographic albums, Vlail’ Kaznacheev Papers, Novosibirsk State Regional Science Library, Novosibirsk (hereafter cited as **NGONB**).

After the first human acclimatization conferences, physicians based in Moscow emphasized that the project of human acclimatization would fail without Indigenous-style fur clothing. However, this immediately revealed an internal conflict within the project, namely the tension between practical needs and colonial hygiene biases. Iosif A. Arnol’di (1899–1979), a renowned Soviet labor hygienist who conducted medical field research in Taymyr, stated in 1962 that ‘although the clothing worn by the indigenous inhabitants of the Arctic features by its high thermal insulation properties, it is very cumbersome and wearing it with fur inside without underwear is extremely unhygienic’^68^ (see Figure 1). His understanding of acclimatization was driven by various factors such as ‘housing conditions, nutrition, clothing, job organization, way of life, etc.’, all of which needed to be adapted to Soviet standards.^69^ This meant that Iosif Arnol’di, like many other acclimatization scholars, placed the imagined ideal Arctic dweller, *Homo Polaris*, within Soviet contexts but detached it from the practicalities of Arctic life. Despite this, scholars continued to debate the balance between Indigenous and Soviet influences in designing Northern or polar clothing almost until the collapse of the Soviet Union. Iosif S. Kandror (1907–2003) followed the lead of his North American colleagues in advocating for the adaptation of Indigenous clothing to meet the needs of Soviet newcomers, recognizing that metropolitan clothing inventions could not meet the requirements of the Arctic. He drew examples from Indigenous Chukchi and Siberian Yupik (or Eskimo in Russian), among whom he conducted his medical field research, to highlight the importance of their ‘traditional’ clothing for modern/Soviet ways of living in the North,^70^

Indeed, all northern peoples use clothing that is unsurpassed in its heat-shielding properties (up to 14 CLO) […] It is interesting that the centuries-old experience of the [I]ndigenous peoples of the North fully meets these [modern] requirements.^71^

The same applied to the ‘traditional’ nomadic dwellings, some features of which scholars saw as potential elements in future mobile housing for fly-in/fly-out workers (R. *vakhtoviki*).^72^ Iosif Kandror mentioned that circular-shaped houses would be more suitable for the Arctic climate compared to the rectangular houses commonly used by settlers.^73^ However, implementing such changes would require a complete overhaul of Soviet planning and industry, making it unlikely to materialize. Scholars who recognized the potential of adapting Indigenous ways of living for settlers still struggled to detach themselves from the settler-colonial ideologies ingrained in the acclimatization project. Reflecting on the acclimatization of Indigenous peoples, Iosif Kandror summarized the debates among Arctic social and medical scholars since the 1950s. He argued that the concept of ‘population acclimatization’ closely resembled the concept of ‘sedentarization’ (R. *osedanie naselenii͡a*) in newly developed regions.^74^ As one can judge from a large collection of published ethnographic reports to the Soviet Communist Party bodies,^75^ the discursive alliance with the state-sponsored program of sedentarization of Indigenous communities was a strategy which promised to make one's research politically actual and personally safe.

After all the discussions and confrontations with dominant political and academic ideologies, the scholars of human acclimatization published professional and public brochures on how to adapt to the northern environment. Initially, during the war era, medical doctors insisted on survival in the North through any means necessary, including practices shared by local and Indigenous communities. However, in the 1960s, a new wave of hygienic discourse emerged, limiting this diversity to sporadic and obvious medical recommendations. These recommendations included wearing warm socks and shoes while working on the permafrost, insulating windows and doors before winter arrives, cold water hardening, providing indoor artificial insulation for children during the polar nights, and more. Additionally, during this time, some large state organizations constructed indoor ‘winter gardens’ as artificial oases of ‘Russian’ climate zones, where workers could relax after their shifts. Despite some attempts and public discussions, Indigenous knowledge and practices were officially positioned beyond the accepted and applied hygienic and nutritional rules.

## II.

Despite the presence of a few Northern acclimatization laboratories^76^ in the North and Siberia, Moscow and Leningrad remained the two primary centers for acclimatization studies. These centers actively utilized data collected by scholars during their medical field expeditions or obtained from the northern laboratories. However, the 20th Congress of the Communist Party, which declared a shift in economic and industrial politics toward Siberia, brought significant changes to the geography of scientific research. This shift was exemplified by the emergence of Novosibirsk as a new Soviet scientific center. In 1957, the Siberian Branch of the Soviet Academy of Sciences was established, followed by the establishment of the Branch of the Soviet Academy of Medical Sciences in 1970.^77^

The ‘father’ of Akademgorodok, Mikhail A. Lavrent’ev (1900–1980), envisioned the new development of Siberia as a socio-technological experiment where human presence was intended to be limited to a small group of engineers controlling the machines.^78^ However, despite Mikhail Lavrent'ev’s technocratic dreams, the North and Siberia could not overlook the fundamental importance of human resources and, consequently, medical care. The works on human acclimatization published in the mid-twentieth century became a new source of inspiration for Siberian medical science.^79^

In 1971, Vlail’ P. Kaznacheev (1924–2014),^80^ the Rector of Novosibirsk Medical Institute, initiated his own acclimatization project in Siberia at the Institute for Clinic and Experimental Medicine, which he established in 1971 under the Siberian Branch of the Soviet Academy of Medical Sciences. From the beginning, the project was intertwined with the overarching idea of Novosibirsk Akademgorodok^81^ and aimed to assist the Soviet state in transforming Siberia and the North into a closely connected network of industrial cities, oil and gas fields, and labor migrants. These migrants, in turn, had to adapt their bodies to the environment of Siberia and the North, which was previously unfamiliar to them.^82^ The Novosibirsk acclimatization project introduced new dimensions to existing scholarship by combining acclimatization studies based on settler-colonial ideologies with eclectic and speculative constructions derived from Soviet philosophy. Consequently, the imagined *Homo Polaris* became epistemically integrated into comprehensive theoretical frameworks but gradually distanced from Siberian Indigenous ways of knowing.

One of the reasons behind these transformations was science diplomacy during the Cold War, particularly the role of the first ecology-oriented discussions^83^ that brought acclimatization studies and the figure of Vlail’ Kaznacheev to prominence within the Soviet Union and beyond.^84^ The establishment of Vlail’ Kaznacheev’s institute coincided with the launch of the UNESCO-based ‘Man and the Biosphere’ program in 1971.^85^ Two years later, the Scientific Committee on Biosphere Issues at the Soviet Academy of Sciences recognized Vlail’ Kaznacheev’s institute as one of the leading institutions in the North and Siberia for studying human acclimatization.^86^ Despite organizing numerous seminars and conferences with Western scholars to assert the primacy of his institute in acclimatization studies, many of his American and European colleagues found his ideas obscure.^87^

**Figure 2.**
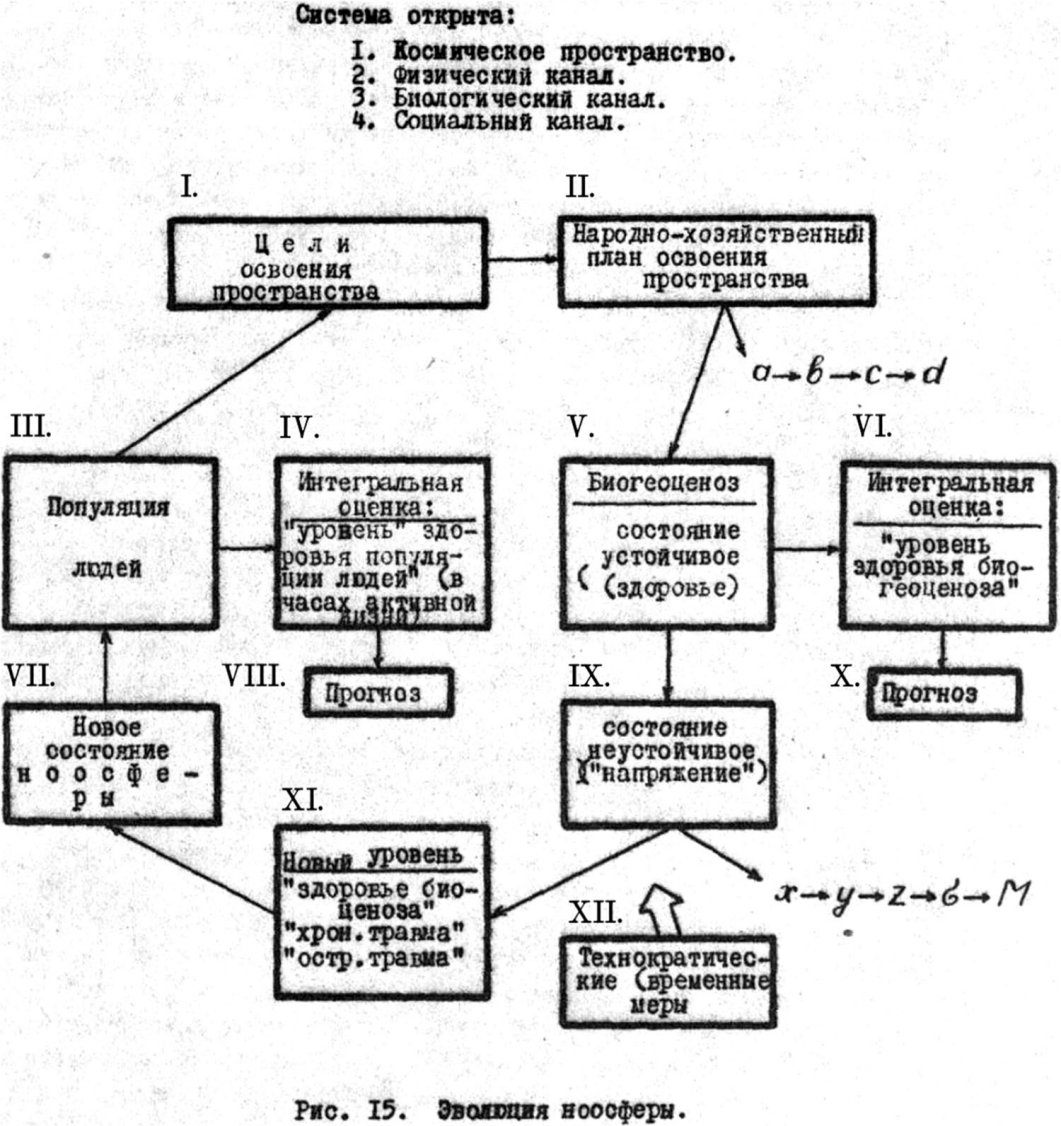
*The Evolution of Noosphere* (Roman numerals added by me – D.A.). I. Goals of the development of [Siberian] space; II. Plan of the national economy for the development of [Siberian] space; III. Human population; IV. Integral estimation: health ‘level’ of the human population measured in hours of active life; V. Biogeocenosis. The steady state (health); VI. Integral estimation: ‘health level of the biogeocenosis’; VII. A new state of noosphere; VIII. Prognosis; IX. The unsteady state (‘tension’); X. Prognosis; XI. A new level of the ‘health of biogeocenosis’, ‘permanent injury’, ‘acute injury’; XII. Technocratic (temporary) actions.

In his quest for recognition in acclimatization studies, Vlail’ Kaznacheev shifted his research toward global theoretical frameworks, with the key word ‘system’ featuring prominently in his writings. As historians and sociologists of knowledge in the late Soviet Union affirm, the term ‘system’ permeated various scientific fields, including human acclimatization, and played a role in maintaining and advancing different forms of knowledge governance.^88^ To illustrate these transformations in the field of acclimatization studies, we can examine one of the diagrams reproduced by Vlail’ Kaznacheev in his early works and later republished in some of his books.^89^ The diagram, titled ‘Evolution of Noosphere’ (Figure 2), visualizes the ‘system’ that encompasses ‘outer (cosmic) space’ (1), a ‘physical channel’ (2), a ‘biological channel’ (3), and a ‘social channel’ (4).

The depicted channels are located at the intersections of the Soviet and the global. By drawing upon the concept of the noosphere, described by Soviet geochemist Vladimir I. Vernadskiy^90^ as a sphere of thought enveloping the entire globe, Vlail’ Kaznacheev referred to the planetary scale of his research project. Interestingly, this conceptualization likely posed a challenge for understanding Vlail’ Kaznacheev’s ideas in the West. Furthermore, the global vision of his scheme was tightly intertwined with the Soviet plan for the development of the North, which is depicted at the top of the diagram (I and II). This positioning caused the noosphere to revolve around the Soviet Union and the North. Simultaneously, the well-being and sustainability of Soviet settlers in the North relied on the ‘health’ of the biogeocenosis, a term that Soviet scholars used as a Russian synonym for ecosystem. Vlail’ Kaznacheev likely adopted this term from the writings of the renowned Soviet geneticist Nikolai V. Timofeev-Resovskiy (1900–1981), who visited Novosibirsk to deliver lectures on genetics.^91^ It is worth noting that Vlail’ Kaznacheev’s planetary modeling excluded Indigenous peoples from the diagram. In other words, the epistemology represented by the depicted arrows, boxes, and numbers was implicitly dominated by Siberian and Northern settlers, who were the driving force behind the new Soviet industrial expansion to Siberia and even beyond its earthly borders.

Despite the diagram’s obscurity, it was utilized as a research model by Vlail’ Kaznacheev’s colleagues. Modified versions of the diagram could be found in reports on various topics, such as the relationship between human cells and solar radiation, compiled by physicians from the Norilsk Laboratory of Polar Medicine.^92^ This laboratory was established in October 1973 as a partner of Vlail’ Kaznacheev’s institute and focused entirely on human acclimatization in the Taymyr peninsula and its capital, Norilsk.^93^ Surviving laboratory reports indicate that the scholars developed the concept of acclimatization as an interconnected process involving human and animal bodies, climate, and diseases.^94^ This approach went beyond the confines of the laboratory building, transforming the entire peninsula into a vast laboratory space – a Siberian equivalent to what historian Helen Tilley has observed about Africa, where ‘virtually every territory served a space for biomedical experimentation on a massive scale’^95^. The members of the Norilsk laboratory examined patients in city clinics and traveled across the peninsula to work with Indigenous peoples (such as Dolgans, Nganasans, Evenkis, Nenetses, and Enetses) and collect swabs and tests for further analysis in the laboratory. The researchers also analyzed medical in-patient cards and other Soviet official documents containing personal information about their patients, which allowed doctors to confidently categorize them as either ‘migrants’ or ‘Indigenous’.^96^ Data extracted from Indigenous patients has always been considered a valuable resource for the further development of settler colonial medicine. The documented measurements of Indigenous and settler bodies have primarily served as a resource for building theoretical constructions rather than proposing and applying practical steps, as we saw in the documents regarding the acclimatization studies in the 1960s. The practical side of the research in the 1980s, as we may speculate, was largely limited to recommendations that medical doctors should provide to their patients.

In their research on the optimal acclimatization of settlers, Vlail’ Kaznacheev and his colleagues developed another biopolitical classification of the Arctic human population, adapting English terms from the realm of sports discourse. They divided the Arctic human population into three categories: ‘sprinters’ (R. *sprintery*), ‘stayers’ (R. *staĭery*), and ‘mixes’ (R. *miksty*).^97^ Vlail’ Kaznacheev argued that the bodies of ‘sprinters’ could adapt to the unfamiliar Arctic environment in the short term but struggled in the long run. The second category, ‘stayers,’ were resilient to the long-term pressures of the unfamiliar environment but faced difficulties in the short term. The ‘mixes’ exhibited a combination of characteristics from the other two categories. This simple framework was applied to all the cases Vlail’ Kaznacheev worked on. Soon after devising this model, he stated that all Arctic and Siberian Indigenous peoples primarily belonged to the ‘stayers’ category due to their extensive history of adaptation in the North and the process of ‘natural selection’, which made them the most acclimatized to the local environment. To a certain extent, the classification may resonate with what we see in GULag documents and colonial histories of medicine.

Vlail’ Kaznacheev’s concept of *Homo Polaris*, or as he referred to it, a model of adaptation,^98^ represented the ‘stayers’ category in his classification. This model aimed to withstand biological and environmental ‘tensions’ (R. *napri͡azhenie*)^99^ until reaching the necessary level of acclimatization for comfortable life in the North. To achieve this, Vlail’ Kaznacheev even considered keeping migrants in the North and Siberia during their holidays and vacations. He partially sought to revive the projects of the 1930s, when Arctic sanatoria were envisioned as hubs for human-nature infrastructure, enabling migrants to develop a deeper connection with the Northern/Siberian environment.^100^ Ultimately, the ‘stayers’ and their descendants could potentially become the ‘new indigenous people’, a topic extensively discussed by Soviet scholars during their meetings.

The role of Siberian and Northern Indigenous peoples and their knowledge was ambiguous. Despite his desire to engage in fieldwork and learn more about Indigenous ways of living, Vlail’ Kaznacheev likely understood that the official acceptance of non-settler colonial ideologies and practices could pose a threat, potentially jeopardizing his Institute and the network of his laboratories.^101^ His inclination toward scholastic theorizing of the human acclimatization process may have been a response to these restrictions and prejudices. However, the system theory he skillfully adopted to human acclimatization turned out to be a means of promoting it far beyond the Arctic and Siberia. In his interviews, Vlail’ Kaznacheev advocated for training Soviet cosmonauts in the Arctic Siberia, as he believed the climate there had many similarities with outer space. And indeed, in the 1980s, some Soviet cosmonauts were trained in the Arctic. According to published memoirs of their medical trainers, their medical programs mirrored the discussions held by human acclimatization scholars.^102^

The history of Soviet research in the Antarctic also shows the degree to which the human acclimatization discourse developed in the works of polar medical doctors. They cited the books and articles of Grigoriy Danishevskiy, Iosif Kandror, Iosif Arnol’di, Vlail’ Kaznacheev and others working in the Arctic.^103^ In other words, settler colonial imaginaries and ideas were transferred to other cold areas as Soviet influence expanded beyond the country’s borders. It supported Vlail’ Kaznacheev’s argument, as visualized in his chart, which positioned the Soviet Union on a planetary scale.

However, Vlail’ Kaznacheev’s scientific language and practices, which had become well-acclimatized to the Soviet political environment, faced certain challenges. By the mid-1980s, the administration of the Soviet Academy of Sciences began expressing skepticism toward some of Vlail’ Kaznacheev’s experiments and his references to non-scientific theories in his works.^104^ This skepticism arose from his incorporation of ideas related to extrasensory perception in Siberian experimental medicine.^105^ Even his medical works, dedicated to some aspects of human acclimatization, tended to be very general and speculative. This made them difficult to read and, more importantly, to understand how to apply. As a result, he quickly found himself excluded from conventional academic circles, and many of his works were regarded as eccentric episodes in the history of Siberian science. The large-scale and well-funded studies on human acclimatization in Siberia and the North began to lose momentum.^106^

### * * *

In this article, I have attempted to trace the intellectual journey of the Arctic human acclimatization project, from its initial carceral experiments to the late Soviet theoretical constructions. Despite the unequal representation of voices involved in the project – medical doctors, administrators, Indigenous and settler colonial communities – this path remained a two-way road. On one hand, throughout its thirty-year history, the project aimed to create a ‘new indigenous population’ in the North, with the intention of settling in the region and becoming the primary labor force. On the other hand, scientists could not help but be amazed by the successful history of survival of Indigenous peoples in the North under various political regimes. These paths coincided with the ‘Sovietization’ of Indigenous ways of life, which did not align with the expected metropolitan standards of hygiene and everyday practices, and the failed attempts of ‘Indigenization’ of settlers who found themselves in a highly unfamiliar environment.

This journey allows us to uncover the narratives and ideologies that accompanied the history of the human acclimatization project and the very idea of the ‘ideal’ inhabitants, whom I refer to in this article as *Homo Polaris*. Deeply rooted in pre-Soviet settler-colonial ideas, the late Soviet medical doctors and administrators were unable to free themselves from that legacy or even critique it. To understand these intertwined genealogies, I examined the ideas and practices of Soviet human acclimatization scholars, with particular attention given to Grigoriy Danishevskiy and Vlail’ Kaznacheev. Despite their different life trajectories and belonging to two different time periods, both contributed to establishing a medical settler colonial project that bore similarities to documented examples of settler colonial science in North America. However, one of the main differences of the Soviet project was the so-called affirmative action politics in the country, which, by the 1960–1980s, solidified into a network of ‘houses of culture’ and supported initiatives to maintain and promote ‘traditional cultures’.

However, like many other settler colonial projects, the human acclimatization in Siberia and the North did not achieve the envisioned success on paper. It encountered numerous difficulties in terms of administration and implementing Indigenous knowledge and practices to meet the settlers’ needs. Medical doctors and engineers either lacked the ability or simply did not want to challenge the prevailing political ideologies. Consequently, the necessity to adapt settlers’ clothing and housing to Indigenous experiences was realized either partially or replaced by speculative theoretical constructions that drew from Soviet philosophy, sociology, genetics, and the ideas of even extrasensory perception. The projected *Homo Polaris*, or rather the set of ideas that I embrace with this term, thus evolved into an abstract theoretical concept, and faced harsh criticism from the Soviet Academy of Sciences.

Nevertheless, due to the administrative skills of medical administrators like Vlail’ Kaznacheev, the idea of human acclimatization in Siberia and the North managed to gain recognition among Antarctic polar workers and Soviet cosmonauts in metropolitan offices. Late Soviet human acclimatization scholars’ works circulated within these circles until the collapse of the Soviet Union, creating an impression of success for the project.

Regardless of how scholars and administrators imagined or regulated human acclimatization, settlers from the European part of the Soviet Union were able to adapt to Arctic lifestyles, which bore resemblances to some practices of Indigenous peoples.^107^ As field ethnographers have documented,^108^ this adaptation occurred through everyday practices outside the industrial cities that settlers projected and built. These practices, such as hunting, fishing, berry picking, and other outdoor activities, as well as friendships and interethnic marriages, brought settlers closer to Indigenous and local communities.^109^ They did not form a homogeneous collective body as the Soviets had planned, but the emerging intersections of their practices were documented in some publications by field ethnographers. Additionally, Rémy Rouillard, a Canadian anthropologist who conducted extensive ethnographic fieldwork among Arctic Nenets, was surprised to find that his interlocutors used terms from the human acclimatization scholarship in their everyday conversations.^110^ These terms likely entered their vocabulary through Russian mass media as an explanation for the biological and psychological challenges newcomers might face in the North. During my own fieldwork among Yamal and Kolguev Nenets, I also encountered similar conversations about the somatic differences between Indigenous peoples and settler colonizers in their reactions to polar nights/days and the low winter temperatures.

Parallel to these vibrant conversations in the tundra, the post-Soviet life of the project remains largely reliant on the achievements of Soviet scholars, as one could read in publications of a few researchers across the Russian North and Siberia continuing to work on the ideas of human acclimatization today.

## Notes

1 J.R. McNeill and Peter Engelke, *The Great Acceleration: An Environmental History of the Anthropocene since 1945* (Cambridge, MA: Harvard University Press, 2016); Andy Bruno, *The Nature of Soviet Power: An Arctic Environmental History* (New York: Cambridge University Press, 2016); Andy Bruno, ‘Studying the Siberian Anthropocene: An Introduction’, *The Soviet and Post-Soviet Review* 48, no. 3 (November 12, 2021): 257–61, https://doi.org/10.30965/18763324-bja10044.

2 Lit. *Chief Directorate of Correctional Labor Camps.* See Anne Applebaum, *Gulag: A History of the Soviet Camps* (London: Penguin Books, 2012). On the legacy of GULag in the North see Alan Barenberg, *Gulag Town, Company Town: Forced Labor and Its Legacy in Vorkuta* (New Haven and London: Yale University Press, 2014); Yuri V. Kanev, *Vaĭgachskiĭ tranzit* (Nar’i͡an-Mar, 2008).

3 On the legacy of early Soviet colonization see in Tatiana V. Naumova, ‘Kulʹtbazy kak ochagi sovetskoĭ kulʹtury v tundre’, *Nauchnyi vestnik I͡Amalo-Nenet͡skogo avtonomnogo okruga*, no. 1(78) (2013): 73–8; Eva Toulouze, Laur Vallikivi, and Art Leete, ‘The Cultural Bases in the North. Sovietisation and Indigenous Resistance’, in *Ethnic and Religious Minorities in Stalin’s Soviet Union: New Dimensions of Research*, ed. Andrej Kotljarchuk and Olle Sundström, vol. 5, Northern Studies Monographs (Stockholm: Södertörns högskola, 2017), 199–224.

4 See Ivan Sablin and Maria Savelyeva, ‘Mapping Indigenous Siberia: Spatial Changes and Ethnic Realities, 1900–2010’, *Settler Colonial Studies* 1, no. 1 (January 1, 2011): 77–110, https://doi.org/10.1080/2201473X.2011.10648802; Vladimir A. Isupov, ed., *Demograficheskai͡a istorii͡a Zapadnoĭ Sibiri (konet͡s XIX – XX vv.)* (Novosibirsk: Izdatel’stvo SO RAN, 2017).

5 The terms acclimatization and adaptation were often used interchangeably. I use them interchangeably in the present paper as well, although scholars in North America preferred to make a distinction specifically in the context of genetics, while considering them synonymous in cultural and social contexts. ‘Adaption’ often refers to ‘genetic adaptation in which an adaptive genotype has emerged through the process of natural selection’, while ‘acclimatization’ refers to ‘nongenetic adjustments to the environment during the lifetime of the individual’. See Joseph K. So, ‘Human Biological Adaptation to Arctic and Subarctic Zones’, *Annual Review of Anthropology* 9, no. 1 (1980): 64.

6 See John McCannon, *Red Arctic: Polar Exploration and the Myth of the North in the Soviet Union, 1932–1939* (Oxford: Oxford University Press, 1998); Paul R. Josephson, *The Conquest of the Russian Arctic* (Cambridge, MA: Harvard University Press, 2014).

7 See Per Högselius, *Red Gas: Russia and the Origins of European Energy Dependence* (New York: Springer, 2012).

8 See a brief ethnographic account on the human acclimatization project in the Russian North today in Rémy Rouillard, ‘Foreign Bodies in the Russian North: On the Physiological and Psychological Adaptation of Soviet Settlers and “Oil Nomads” to the Oil-Rich Arctic’, in *Northern Sustainabilities: Understanding and Addressing Change in the Circumpolar World*, ed. Gail Fondahl and Gary N. Wilson, Springer Polar Sciences (Cham: Springer International Publishing, 2017), 163–76, https://doi.org/10.1007/978-3-319-46150-2_13.

9 In the history of Soviet medical sciences, that pressure became entwined with the social hygiene project actively promoted in the early years of Soviet power. See Susan Gross Solomon and John F. Hutchinson, eds., *Health and Society in Revolutionary Russia* (Bloomington and Indianapolis: Indiana University Press, 1990).

10 It is worth mentioning that Soviet scholars, engineers, and medical doctors also actively got involved in the Antarctic geopolitical competition. See Simone Turchetti et al., ‘On Thick Ice: Scientific Internationalism and Antarctic Affairs, 1957–1980’, *History and Technology* 24, no. 4 (December 1, 2008): 351–76, https://doi.org/10.1080/07341510802357419.

11 Referring to the settler colonizers from the European part of Russia, I aim to avoid the ethnic connotation of the word ‘Russians’ (R. *russkie*) and instead emphasize the multiethnic composition of the new migrants, who primarily relocated from the European regions and republics of the Soviet Union.

12 See about the relations between the environment and subjectivity in the Soviet North in Andy Bruno, ‘Environmental Subjectivities from the Soviet North’, *Slavic Review* 78, no. 1 (2019): 1–22, https://doi.org/10.1017/slr.2019.7.

13 See about that politics in Yuri Slezkine, ‘The USSR as a Communal Apartment, or How a Socialist State Promoted Ethnic Particularism’, *Slavic Review* 53, no. 2 (1994): 414–52, https://doi.org/10.2307/2501300. See also about the identity politics in North Siberia in David G. Anderson, *Identity and Ecology in Arctic Siberia: The Number One Reindeer Brigade* (Oxford: Oxford University Press, 2000).

14 Grigoriy M. Danishevskiy, *Patologii͡a cheloveka i profilaktika zabolevaniĭ na Severe* (Moscow: Medit͡sina, 1968), 115. Some acclimatization scholars called it a model or a benchmark (R. *ėtalon*). See Vlail’ P. Kaznacheev, *Sovremennye aspekty adaptat͡sii* (Novosibirsk: Nauka, 1980), 145.

15 V.F. Puzanova, ed., *Materialy rasshirennogo plenuma Mezhduvedomstvennoĭ Komissii po problemam Severa*, vol. 5. Kratkoe soderzhanie vystupleniĭ i reshenie plenuma (Moscow: SOPS, 1970), 115.

16 See about the notion of settler colonialism in Lorenzo Veracini, *Settler Colonialism: A Theoretical Overview* (New York: Palgrave Macmillan, 2010); Lorenzo Veracini, ‘“Settler Colonialism”: Career of a Concept’, *The Journal of Imperial and Commonwealth History* 41, no. 2 (June 1, 2013): 313–33, https://doi.org/10.1080/03086534.2013.768099. As historians of science remind us, the human acclimatization projects inherited colonial disparities and biopolitical ideologies that can be traced back to eighteenth-century discussions and practices in metropolitan France, Britain, and their colonies. See David N. Livingstone, ‘Human Acclimatization: Perspectives on a Contested Field of Inquiry in Science, Medicine and Geography’, *History of Science* 25, no. 4 (1987): 359–94; Michael A. Osborne, ‘Acclimatizing the World: A History of the Paradigmatic Colonial Science’, *Osiris* 15 (2000): 135–51; Hans Pols, ‘Notes from Batavia, the Europeans’ Graveyard: The Nineteenth-Century Debate on Acclimatization in the Dutch East Indies’, *Journal of the History of Medicine and Allied Sciences* 67, no. 1 (2012): 120–48.

17 As Patrik Chakrabarti indicates, the shift from ‘colonial medicine’ as carrying the bodies of colonizers to the ‘civilizing mission’ of medicine occurred in the late nineteenth century. See Pratik Chakrabarti, *Medicine and Empire: 1600–1960* (Palgrave MacMillan, 2014).

18 Alexander Morrison, ‘Russian Settler Colonialism’, in *The Routledge Handbook of the History of Settler Colonialism*, ed. Edward Cavanagh and Lorenzo Veracini (London and New York: Routledge, 2017), 313–26. Interestingly, the leaders of the late 19th-century Siberian regionalism movement (R. *Oblastniki*) highlighted the historical significance of ‘mixing’ peoples (R. *smeshenie narodov*) in the process of colonization. See Nikolai M. Yadrintsev, *Sibir’ kak kolonii͡a v geograficheskom, ėtnograficheskom i istoricheskom otnoshenii*, 2nd ed. (Saint Petersburg: Izdanie I. M. Sibiri͡akova, 1892). See also about the *Oblastniki* David Rainbow, ‘Siberian Patriots: Participatory Autocracy and the Cohesion of the Russian Imperial State, 1858–1920’ (PhD thesis, New York, New York University, 2013). In the tweentieth century, field ethnographers and linguists, in turn, documented the lives and languages of numerous mixed communities in Siberia and the Far East (Peter P. Schweitzer, Evgeniy V. Golovko, and Nikolai B. Vakhtin, ‘Mixed Communities in the Russian North; Or, Why Are There No “Creoles” In Siberia?’, *Ethnohistory* 60, no. 3 (July 1, 2013): 419–38, https://doi.org/10.1215/00141801-2140749.). However, it is known that the dominant state-sponsored ethnographic discourse has reduced the cultural, language, and ethnic diversity of the region to the list of officially recognized ethnic groups, thereby making the mixed communities invisible (David G. Anderson and Dmitry V. Arzyutov, ‘The Construction of Soviet Ethnography and “The Peoples of Siberia”’, *History and Anthropology* 27, no. 2 (March 14, 2016): 183–209, https://doi.org/10.1080/02757206.2016.1140159; See also Francine Hirsch, *Empire of Nations: Ethnographic Knowledge & the Making of the Soviet Union* (Ithaca: Cornell University Press, 2005).)

19 Alexey Golubev, ‘No Natural Colonization: The Early Soviet School of Historical Anti-Colonialism’, *Canadian Slavonic Papers* 65, no. 2 (May 25, 2023): 190–204, https://doi.org/10.1080/00085006.2023.2199556.

20 In the Soviet North, the politics of Indigenization (R. *korenizat͡sii͡a*) was replaced by the hierarchical model in the late 1930s. Yuri Slezkine ironically phrases that shift as putting Indigenous peoples to be ‘last among equals’. Yuri Slezkine, *Arctic Mirrors: Russia and the Small Peoples of the North* (Ithaca, NY: Cornell University Press, 1994).

21 See Terry D. Martin, *The Affirmative Action Empire: Nations and Nationalism in the Soviet Union, 1923–1939* (Ithaca and London: Cornell University Press, 2001).

22 Bruce Grant, *In the Soviet House of Culture: A Century of Perestroikas* (Princeton, NJ: Princeton University Press, 1995).

23 See Igor’ Stas’, ‘Vozrozhdennye narody: nat͡sional’noe grazhdanstvo indigennogo naselenii͡a Severa v pozdnem stalinizme’, *Ab Imperio* 2020, no. 3 (2020): 157–88, https://doi.org/10.1353/imp.2020.0060. An anthropological account on the life of Russian settler colonial communities in the Far East: see Niobe Thompson, *Settlers on the Edge: Identity and Modernization on Russia’s Arctic Frontier* (Vancouver: University of British Columbia Press, 2009).

24 See Maksym Sviezhentsev, ‘“Phantom Limb”: Russian Settler Colonialism in the Post-Soviet Crimea (1991–1997)’ (Ph.D. dissertation in History, London, ON, The University of Western Ontario, 2020).

25 Veracini, ‘“Settler Colonialism”’, 313–14.

26 Travis Hay, *Inventing the Thrifty Gene: The Science of Settler Colonialism* (Winnipeg: University of Manitoba Press, 2021).

27 M. Trakman, *Perspektivy razvitii͡a zdravookhranenii͡a v Sibiri (1928/29–1932/33 g.)* (Novosibirsk, 1929).

28 See journalist notes of Russian anthropologist Waldemar Bogoras about the Far East: Vladimir G. Bogoraz, ‘Russkie na reke Kolyme’, *Zhiznʹ* 6 (1899): 121.

29 Here I should refer to the classic social history understanding of the concept of *making* as an active process, ‘which owes as much to agency as to conditioning’: Edward P. Thompson, *The Making of the English Working Class* (New York: Vintage Books, 1966), 9.

30 See its history in Kathleen E. Smith, *Moscow 1956: The Silenced Spring* (Cambridge, MA and London: Harvard University Press, 2017).

31 V. Gurevich, ed., *XX s”ezd Kommunisticheskoĭ Partii Sovetskogo Soi͡uza. 14–25 fevrali͡a 1956 g. Stenograficheskiĭ otchet*, vol. I (Moscow: Gosudarstvennoe izdatelʹstvo politicheskoĭ literatury, 1956), 52.

32 See Kristen E. Edwards, ‘Fleeing to Siberia The Wartime Relocation of Evacuees to Novosibirsk, 1941–1943’ (Ph.D. Dissertation in History, Palo Alto, Stanford University, 1996).

33 See the discussions among ‘civil’ medical doctors about the Arctic experimental medicine in the 1930s. A. Bliumenfelʹd, ‘Igarka - poli͡arnai͡a baza Vsesoi͡uznogo Instituta ėksperimentalʹnoĭ medit͡siny pri SNK SSSR’, *Severnai͡a stroĭka*, September 10, 1934.

34 Asif Siddiqi, ‘Scientists and Specialists in the Gulag: Life and Death in Stalin’s Sharashka’, *Kritika: Explorations in Russian and Eurasian History* 16, no. 3 (2015): 557–88.

35 See about the Pechorlag clinics in Svetlana Terent’eva and Tatiana Afanas’eva, *Vremena ne vybirai͡ut … Iz istorii zdravookhranenii͡a Pechorskogo Krai͡a* (Syktyvkar: Komi respublikanskai͡a tipografii͡a, 2012), 19–40. The historian of GULag medical service Boris Nakhapetov points out that the Pechorlag was one of the most terrifying in the whole GULag archipelago Boris A. Nakhapetov, *Ocherki Istorii Sanitarnoĭ Sluzhby GULAGA* (Moscow: ROSSPĖN, 2009), 23–24. On the connections between GULag and non-GULag worlds see Oleg Khlevniuk, ‘The Gulag and the Non-Gulag as One Interrelated Whole’, trans. Simon Belokowsky, *Kritika: Explorations in Russian and Eurasian History* 16, no. 3 (2015): 479–98.

36 These are his books based on the data collected during those years: Grigoriy M. Danishevskiy, *Gipovitaminozy na Severe: ranni͡ai͡a diagnostika, profilaktika i lechenie* (Pechora, 1944); Grigoriy M. Danishevskiy, *Akklimatizat͡sii͡a cheloveka na Severe (s ocherkom kraevoĭ patologii i gigieny)* (Moscow: Medgiz, 1955). See also the official list of diseases of GULag prisoners compiled by the head of GULag medical service in 1942: Nakhapetov, *Ocherki istorii sanitarnoĭ sluzhby GULAGA*, 134–39.

37 It is worth mentioning that the project of polar/Arctic agriculture was actively developed throughout the Soviet North from the early 1930s until the end of the Soviet era.

38 Golfo Alexopoulos, ‘Medical Research in Stalin’s Gulag’, *Bulletin of the History of Medicine* 90, no. 3 (October 27, 2016): 368, https://doi.org/10.1353/bhm.2016.0070; See also Dan Healey, ‘Lives in the Balance: Weak and Disabled Prisoners and the Biopolitics of the Gulag’, *Kritika: Explorations in Russian and Eurasian History* 16, no. 3 (2015): 527–56, https://doi.org/10.1353/kri.2015.0047; Oxana Ermolaeva, ‘Health Care, the Circulation of Medical Knowledge, and Research in the Soviet Gulag in the 1930s’, *East Central Europe* 40, no. 3 (2013): 341–65, https://doi.org/10.1163/18763308-04003008.

39 For example, Lev Zil’ber (1894–1966), a colleague of Grigoriy Danishevskiy, invented an ‘anti-pellagrin’ made from yeast and tundra lichen that saved hundreds of human lives in prisons during the 1930s and later in the Second World War. It is worth mentioning that the official certificate credited the NKVD (*People’s Commissariat for Internal Affairs*, the major Soviet repressive authority from 1934 to 1946) rather than Zil’ber as the preparator inventor. See Lev L. Kiselev and Elena S. Levina, *Lev Aleksandrovich Zil’ber (1894–1966). Zhizn’ v nauke* (Moscow: Nauka, 2004), 244–49.

40 Danishevskiy, *Akklimatizat͡sii͡a cheloveka na Severe (s ocherkom kraevoĭ patologii i gigieny)*, 143.

41 Ibid., 85–87. See also V.L. Vasil’ev, K.A. Turkovskaia, and M.M. Khrennikova, *Prodvizhenie ovoshcheĭ na Kraĭniĭ Sever* (Moscow and Leningrad: Gosudarstvennoe izdatel’stvo kolkhoznoĭ i sovkhoznoĭ literatury, 1935).

42 Applebaum, *Gulag*, 369–79; Golfo Alexopoulos, *Illness and Inhumanity in Stalin’s Gulag* (New Haven and London: Yale University Press, 2017), 62–84.

43 Danishevskiy, *Akklimatizat͡sii͡a cheloveka na Severe (s ocherkom kraevoĭ patologii i gigieny)*, 88–92.

44 See Irina V. Il’ina, *Tradit͡sionnai͡a medit͡sinskai͡a kulʹtura narodov Evropeĭskogo Severo-Vostoka (konet͡s XIX - XX vv.)* (Syktyvkar, 2008); Boris I. Vasilenko, *Narodnai͡a medit͡sina nent͡sev I͡Amala* (Salekhard: Krasnyĭ Sever, 1997).

45 It is worth comparing the Russian/Soviet carceral genealogy to observations made by historians of medicine and environmental historians who write about ‘social’ diseases such as smallpox, typhus, or tuberculosis as legacies of European colonialism. Alfred W. Crosby, *Ecological Imperialism: The Biological Expansion of Europe, 900–1900*, 2nd ed. (Cambridge and New York: Cambridge University Press, 2004); Chakrabarti, *Medicine and Empire*.

46 Vladimir N. Davydov and Veronika V. Simonova, ‘Sobachʹe serdt͡se: antropologii͡a sobakoedenii͡a v postsovetskoĭ ėvenkiĭskoĭ derevne’, *Izvestii͡a laboratorii drevnikh tekhnologiĭ*, no. 6 (2008): 218–19. [Evenkis]

47 Vladimir N. Adaev, ‘Nenet͡skie olenegonnye laĭki Tazovskoĭ tundry’, in *Ab Originale. Arkheologo-ėtnograficheskiĭ sbornik Ti͡umenskogo gosudarstvennogo universiteta*, ed. Natalʹi͡a P. Matveeva, vol. 5 (Ti͡umenʹ: Ti͡umenskiĭ gosudarstvennyĭ universitet, 2013), 130. [Nenetses]

48 Olga Ulturgasheva, ‘Ghosts of the Gulag in the Eveny World of the Dead’, *The Polar Journal* 7, no. 1 (January 2, 2017): 26–45, https://doi.org/10.1080/2154896X.2017.1329256.

49 Terent’eva and Afanas’eva, *Vremena ne vybirai͡ut … Iz Istorii zdravookhranenii͡a Pechorskogo krai͡a*, 41–48.

50 See Ivan A. Perfil’ev, *Kak obespechit’ sebi͡a vitaminami* (Arkhangel’sk: OGIZ Arkhangel’skoe izdatel’stvo, 1941); Aleksandr F. Nikitin, ‘Nauchnye osnovy pitanii͡a v Arktike’, *Priroda*, no. 1–2 (1942): 70–80; Aleksandr F. Nikitin, ‘Arktika i Kraĭniĭ Sever kak moshchnyĭ istochnik pishchevykh resursov dli͡a SSSR’, *Priroda*, no. 5–6 (1942): 70–76. One could also mention some Soviet films on the topic, one of which depicted wartime school expeditions to the Novaya Zemlya archipelago for collecting wild bird eggs: *Letni͡ai͡a poezdka k mori͡u*, 1978, http://www.imdb.com/title/tt1733166/. See about those expeditions: Vladimir N. Bulatov, ‘Shkolʹnai͡a arkticheskai͡a ėkspedit͡sii͡a’, in *Poli͡arnyĭ krug*, ed. A.V. Shumilov (Moscow: Myslʹ, 1986), 175–81.

51 See V. Ya. Chekin, ‘Chto predokhrani͡aet narodnosti kraĭnego severa ot t͡syngi’, *Priroda*, no. 10 (1950): 53. Interestingly, the author advocated for the acceptance of Indigenous culture, specifically the Nenets, regarding the consumption of raw meat and fish (Tundra Nenets *ngay¡a¦bad°*). See about the Nenets food culture Elena T. Pushkareva, *Gastronomicheskie vstrechi : ėtnograficheskie ocherki o kukhne nent͡sev i ne tolʹko … *, 2nd ed. (Salekhard: I͡Amal-Media, 2022). See also Danishevskiy, *Akklimatizat͡sii͡a cheloveka na Severe (s ocherkom kraevoĭ patologii i gigieny)*, 95. To draw a historical parallel, I would like to mention the works of Vilhjálmur Stefánsson on the Inuit exclusively-meat diet, which is also known as the ‘ketogenic diet’. Vilhjalmur Stefansson, *Not by Bread Alone* (New York: Macmillan, 1946); see also Gísli Pálsson, *Travelling Passions: The Hidden Life of Vilhjalmur Stefansson*, trans. Keneva Kunz (Hanover, NH: University Press of New England [for] Dartmouth College, 2005). A decade later, the recommendations to consume raw meat and fish were criticized as a potential threat for helminthiasis. See Iosif A. Arnol’di, *Akklimatizat͡sii͡a cheloveka na Severe i I͡Uge* (Moscow: Medgiz, 1962), 43.

52 P.E. Terletskiy. ‘Problema zaselenii͡a Kraĭnego Severa’, 24 March 1945, Central State Archive of Science and Technological Documentation of Saint Petersburg (hereafter cited as **T͡SGANTD SPb**) R-369/1-4/24.

53 ‘Programma konferent͡sii po voprosam akklimatizat͡sii naselenii͡a v Arktike’, 6 March 1950, T͡SGANTD SPb R-369/1-4/217/154-155 (unclassified). It is worth mentioning that until the late 1960s the Arctic medicine was strongly associated with the Soviet military (‘Plan provedenii͡a nauchnoĭ konferent͡sii po izuchenii͡u medit͡sinskikh problem v Arktike’, T͡SGANTD SPb R-369/1-4/367/236-239 (unclassified)) similar as its North American counterparts. See Matthew Farish, ‘The Lab and the Land: Overcoming the Arctic in Cold War Alaska’, *Isis* 104, no. 1 (March 2013): 1–29, https://doi.org/10.1086/669881; Matthew Wiseman, ‘Unlocking the “Eskimo Secret”: Defence Science in the Cold War Canadian Arctic, 1947–1954’, *Journal of the Canadian Historical Association / Revue de La Société Historique Du Canada* 26, no. 1 (2015): 191–223, https://doi.org/10.7202/1037202ar; Matthew S. Wiseman, ‘The Development of Cold War Soldiery: Acclimatisation Research and Military Indoctrination in the Canadian Arctic, 1947–1953’, *Canadian Military History* 24, no. 2 (November 23, 2015): 127–55.

54 As part of the post-war interest in human acclimatization studies in the North, several expeditions were sent to various parts of the Soviet Arctic under the leadership of Iosif A. Arnol’di. See Grigoriy M. Danishevskiy, *Patologii͡a cheloveka i profilaktika zabolevaniĭ na Severe* (Moscow: Medit͡sina, 1968), 20.

55 Cassandra Cavanaugh, ‘Acclimatization, the Shifting Sciences of Settlement’, in *Peopling the Russian Periphery: Borderland Colonization in Eurasian History*, eds. Nicholas Breyfogle, Abby Schrader, and Willard Sunderland (Routledge, 2007), 169–88.

56 Vladimir L. Naidin, ‘Proveri͡ai͡a gipotezu’, *Znanie-sila*, no. 7 (1968): 34–37. That optimization was made possible due to the establishment of the Commission on Acclimatization and Regional Pathology in the North as early as 1958. See Danishevskiĭ, *Patologii͡a cheloveka i profilaktika zabolevaniĭ na Severe*, 22. This commission was closely linked to the powerful inter-agency Commission on the Problems of the North, which was established in 1954 and was responsible for administering Northern development in the Soviet Union. (See Ekaterina A. Kalemeneva, ‘Smena modeleĭ osvoenii͡a sovetskogo Severa v 1950-e gg. Sluchaĭ Komissii po problemam Severa’, *Sibirskie istoricheskie issledovanii͡a*, no. 2 (2018): 181–200.

57 Alexopoulos, *Illness and Inhumanity in Stalin’s Gulag*, 62.

58 Ibid., 63–6.

59 Danishevskiy, *Akklimatizat͡sii͡a cheloveka na Severe (s ocherkom kraevoĭ patologii i gigieny)*.

60 Alan C. Burton and Otto G. Edholm, *Man in a Cold Environment: Physiological and Pathological Effects of Exposure to Low Temperatures* (London: Edward Arnold Ltd., 1955); Alan Barton and Otto Edkholm, *Chelovek v uslovii͡akh kholoda* (Moscow: Izdatel’stvo inostrannoĭ literatury, 1957). See also Iosif S. Kandror, ed., *Medit͡sina i zdravookhranenie v Arktike i Antarktike. Izbrannye Materialy Konferent͡sii*, vol. 18, Tetradi Obshchestvennogo Zdravookhranenii͡a (Moscow; Geneva, 1962).

61 See Brian Roberts, ‘The Study of Man’s Reaction to a Polar Climate’, *Polar Record* 4, no. 26 (1943): 63–9; MacDonald Critchley, ‘Problems of Naval Warfare Under Climatic Extremes’, *The Military Surgeon* 98, no. 2 (1946): 94–106; Macdonald Critchley, ‘Effects of Climatic Extremes: Critical Review’, *British Journal of Industrial Medicine* 4, no. 3 (1947): 164–90; N. H. Mackworth, ‘Local Cold Acclimatization in Man’, *Polar Record* 8, no. 52 (1956): 13–21.

62 The 1960 Moscow conference marked the first official gathering of all the leading scholars of Arctic acclimatization. See *Trudy nauchnogo soveshchanii͡a po problemam akklimatizat͡sii i pitanii͡a naselenii͡a na Severe*, vol. 6, Problema Severa (Moscow: Izdatel’stvo AN SSSR, 1962). It was followed by three regional conferences held in Murmansk (22–24 June 1961), Archangelsk (14–16 March 1963), and Norilsk (5–8 June 1965). See *Klimat i zdorov’e cheloveka na Kraĭnem Severe: Tezisy dokladov nauchnoĭ sessii Akademii Medit͡sinskikh Nauk SSSR i Ministerstva zdravookhranenii͡a RSFSR v Murmanske. 22–24 Ii͡uni͡a 1961 g.* (Moscow: Medgiz, 1961); S.P. Speranskiy, ed., *Nauchnai͡a konferent͡sii͡a po probleme ‘akklimatizat͡sii͡a i klimatopatologii͡a cheloveka na Severe’ (14–16 Marta 1963 g.).* (Arkhangel’sk, 1963); *Nauchnai͡a sessii͡a, posvi͡ashchennai͡a probleme ‘Trud i zdorov’e cheloveka na Kraĭnem Severe’ (5–8 Ii͡uli͡a 1965 g.). Materialy k nauchnoĭ sessii* (Noril’sk, 1965).

63 Danishevskiy, *Akklimatizat͡sii͡a cheloveka na Severe (s ocherkom kraevoĭ patologii i gigieny)*, 225.

64 The Red Corner (R. *Krasnyĭ ugolok*) in the early Soviet Union referred to a section of public space dedicated to *likbez* (short for ‘likvidat͡sii͡a bezgramotnosti’, meaning ‘elimination of illiteracy’) through the use of visual and textual propaganda materials.

65 Here, he is most likely referring to the Nenets or Komi peoples, whose traditional nomadic dwelling is called a ‘chum’ in Russian.

66 Danishevskiy, *Patologii͡a cheloveka i profilaktika zabolevaniĭ na Severe*, 81, 114.

67 See Ibid., 90–114. See also about the notion of *kul’turnost’* in the Soviet public discourse Vadim Volkov, ‘The Concept of Kul’turnost’: Notes on the Stalinist Civilizing Process’, in *Stalinism: New Directions*, ed. Sheila Fitzpatrick (London: Routledge, 2000), 210–30.

68 Iosif A. Arnol’di, *Akklimatizat͡sii͡a cheloveka na severe i i͡uge* (Moscow: Medgiz, 1962), 40.

69 Ibid., 5.

70 For example, the Institute for Polar Agriculture based in Norilsk provided its field researchers with equipment that included partially Indigenous fur garments such as the *malitsa* (Nenets *maly°cya*), a fur overcoat, or the *unty* (Evenki's *unta*), fur footwear. See Norilsk City Archive, Norilsk (hereafter cited as **NCA**), R-12/1/8: 93.

71 Iosif S. Kandror, *Ocherki po fiziologii i gigiene cheloveka na Kraĭnem Severe* (Moscow: Medit͡sina, 1968), 84, 248.

72 Nataliia A. Saprykina, *Mobil’noe zhilishche dli͡a Severa* (Leningrad: Stroĭizdat, 1986). See about *vakhtoviki* in the Russian North in Natalya N. Simonova, *Adaptat͡sii͡a k rabote vakhtovym metodom v ėkstremalʹnykh uslovii͡akh Kraĭnego Severa* (Arkhangelsk: Severnyĭ (Arkticheskiĭ) federalʹnyĭ universitet imeni M.V. Lomonosova, 2014); Mattias Spies, ‘Potentials for Migration and Mobility among Oil Workers in the Russian North’, *Geografiska Annaler. Series B, Human Geography* 91, no. 3 (2009): 257–73.

73 Kandror, *Ocherki po fiziologii i gigiene cheloveka na Kraĭnem Severe*, 255.

74 Ibid., 264.

75 See Zoia P. Sokolova and Elena A. Pivneva, eds., *Ėtnologicheskai͡a ėkspertiza: narody Severa Rossii: 1956–1958 gody* (Moscow: Institut ėtnologii i antropologii im. N.N. Miklukho-Maklai͡a RAN, 2004); Zoia P. Sokolova and Elena A. Pivneva, eds., *Ėtnologicheskai͡a ėkspertiza: narody Severa Rossii: 1959–1962 gody* (Moscow: Institut ėtnologii i antropologii im. N.N. Miklukho-Maklai͡a RAN, 2005); Zoia P. Sokolova and Elena A. Pivneva, eds., *Ėtnologicheskai͡a ėkspertiza: narody Severa Rossii* (Moscow: Institut ėtnologii i antropologii im. N.N. Miklukho-Maklai͡a RAN, 2006).

76 Vladimir P. Pashchenko, *Problemna laboratorii͡a po izuchenii͡u akklimatizat͡sii cheloveka na Kraĭnem Severe. Iz istorii nauchnykh podrazdeleniĭ AGMI-AGMA-SGMU* (Arkhangelsk, 2001).

77 See the history of Novosibirsk Akademgorodok Paul R. Josephson, *New Atlantis Revisited: Akademgorodok, the Siberian City of Science* (Princeton University Press, 1997).

78 Vlail’ P. Kaznacheev, ‘Mysli i chuvstva na zakate intellektual’no t͡sentra t͡sivilizat͡sii’, in *Gorodok.Ru: Novosibirskiĭ akademgorodok na poroge tret’ego tysi͡acheletii͡a. Vospominanii͡a, razmyshlenii͡a, proekty.* (Novosibirsk, 2003), 228–29. See also Ksenia Tatarchenko, ‘Calculating a Showcase: Mikhail Lavrentiev, the Politics of Expertise, and the International Life of the Siberian Science-City’, *Historical Studies in the Natural Sciences* 46, no. 5 (2016): 592–632. Lavrent’ev's technocratic settler colonial utopia in Siberia was situated within a broader context of cybernetics and later system theory. See about Soviet cybernetics in Slava Gerovitch, *From Newspeak to Cyberspeak: A History of Soviet Cybernetics* (Cambridge, MA and London: MIT Press, 2002).

79 V.P. Kaznacheev, ‘30 let Akademii Medit͡sinskikh nauk SSSR’, 23–24 April 1975, State Archive of Novosibirsk Region, Novosibirsk (hereafter cited as **GANO**), R-2063/1/116/23.

80 Kaznacheev’s audio recording, 26 May 2007, Kaznacheev Papers, NGONB.

81 Watch the following documentaries, *Chelovek i Sever*, Documentary, 1982, https://www.net-film.ru/en/film-48896/; *Chelovek i Sever*, Documentary, 1985, https://www.youtube.com/watch?v=At4bOlN0ljo; *Zemli͡a rodnai͡a*, Documentary, 1986, https://www.youtube.com/watch?v=C4lx0kW1ZqU; *Akademik Vlailʹ Kaznacheev. Monologi*, Documentary, 2014, https://www.youtube.com/watch?v=7KPVuyhIYuA.

82 It is worth noting that at the same time, the concept of techno-geological ‘oases’ as socio-techno-geological hubs gained popularity not only among scholars but also among engineers and administrators in both metropolitan and Siberian regions. See about it, Samuil V. Slavin, *Promyshlennoe i transportnoe osvoenie Severa SSSR* (Moscow: Ėkonomizdat, 1961).

83 See Paul Warde, Libby Robin, and Sverker Sörlin, *The Environment: A History of the Idea* (Baltimore: Johns Hopkins University Press, 2018).

84 It is worth mentioning that Vlail’ Kaznacheev actively promoted his research through international collaborations with various Western scholars. In 1976, his Institute hosted the 4th Symposium for Circumpolar Health. See Neil J. Murphy, ‘The Roundtrip to Fairbanks: The Circumpolar Health Movement Comes Full Circle, Part II’, *International Journal of Circumpolar Health* 72, no. 1 (January 31, 2013): 21608, https://doi.org/10.3402/ijch.v72i0.21608.

85 Simone Schleper, ‘Conservation Compromises: The MAB and the Legacy of the International Biological Program, 1964–1974’, *Journal of the History of Biology* 50, no. 1 (2017): 133–67.

86 ‘Problemy fundamental’nyĭ issledovaniĭ biosfery Evropeĭskogo Severa i Sibiri’, [1979], Archive of Russian Academy of Sciences, Moscow (**ARAN**) 2092/1/1/116. Moreover, Vlail’ Kaznacheev was aware of the work done within the preceding International Biological Program (IBP, 1964–1974), which included a Human Adaptability Project with strong links to ecosystem ecology and some epigenetic ideas. The Soviet group of the Project was led by Zoi͡a I. Barbashova who worked in the Archangelsk acclimatization laboratory.

87 So, ‘Human Biological Adaptation to Arctic and Subarctic Zones’. Vlail’ Kaznacheev’s colleagues also recollected that the terminology he had used remained quite vague to them. See Ol’ga Loshkareva, ‘Akademik V.I. Konenkov: “Immunitet u kazhdogo svoĭ”’, Nauka iz pervykh ruk, July 12, 2022, http://scfh.ru/news/akademik-v-i-konenkov-immunitet-u-kazhdogo-svoy/.

88 Eglė Rindzevičiūtė points out the links between the epistemic design of system theory and its political implications, which she refers to as system-cybernetic governmentality. See Eglė Rindzevičiūtė, *The Power of Systems: How Policy Sciences Opened Up the Cold War World* (Cornell University Press, 2016).

89 Vlail’ P. Kaznacheev, *Biosistema i adaptat͡sii͡a* (Novosibirsk, 1973), 52; Kaznacheev, *Sovremennye aspekty adaptat͡sii*, 41.

90 J. Oldfield and D. J. B. Shaw, ‘V.I. Vernadsky and the Noosphere Concept: Russian Understandings of Society-Nature Interaction’, *Geoforum* 37, no. 1 (2006): 145–54; George M. Young, *The Russian Cosmists: The Esoteric Futurism of Nikolai Fedorov and His Followers* (New York: Oxford University Press, 2012), 163–71.

91 See Douglas R Weiner, *A Little Corner of Freedom: Russian Nature Protection from Stalin to Gorbachev* (Berkeley, Los Angeles, London: University of California Press, 1999), 386–99.

92 V.I. Khasnulin, ‘Vyi͡asnenie roli klimato-geograficheskikh faktorov v prot͡sesse adaptat͡sii i v vozniknovenii ri͡ada zabolevaniĭ v uslovii͡akh Zapoli͡ar’i͡a’, 1980, NCA, R-56/3/4/48; V.I. Khasnulin, ‘Vyi͡avlenie spet͡sificheskikh osobennosteĭ vozniknovenii͡a i techenii͡a ostrykh i khronicheskikh infekt͡sionnykh zabolevaniĭ v sisteme organov dykhanii͡a u korennogo i prishlogo naselenii͡a Kraĭnego Severa … ’, 14 May 1979, NCA, R-56/3/5.

93 An overview of the collection ‘Laboratorii͡a poli͡arnoĭ medit͡siny SO AMN SSSR’, NCA, R-56. Kaznacheev also actively collaborated with Archangelsk laboratory. See Pashchenko, *Problemna laboratorii͡a po izuchenii͡u akklimatizat͡sii cheloveka na Kraĭnem Severe. Iz Istorii nauchnykh podrazdeleniĭ AGMI-AGMA-SGMU*, 15.

94 B.S. Yudin, ‘Zoologo-parazitologicheskie aspekty adaptat͡siĭ cheloveka k uslovii͡am Severa’, 26 February 1979, NCA, R-56/3/3; V. I. Khasnulin, ‘Vyi͡asnenie roli klimato-geograficheskikh faktorov v prot͡sesse adaptat͡sii i v vozniknovenii ri͡ada zabolevaniĭ v uslovii͡akh Zapoli͡ar'i͡a’, 1980, NCA, R-56/3/4; V.I. Khasnulin, ‘Vyi͡avlenie spet͡sificheskikh osobennosteĭ vozniknovenii͡a i techenii͡a ostrykh i khronicheskikh infekt͡sionnykh zabolevaniĭ v sisteme organov dykhanii͡a u korennogo i prishlogo naselenii͡a Kraĭnego Severa … ’, 14 May 1979, NCA, R-56/3/5; V.N. Nikolaev, et al. Vlii͡anie geofizicheskikh i meteorologicheskikh faktorov vneshneĭ sredy na organizm cheloveka v uslovii͡akh Zapadnoĭ Sibiri (programma Globėks-80), 1980, NCA, R-56/3/6.

95 Helen Tilley, *Africa as a Living Laboratory: Empire, Development, and the Problem of Scientific Knowledge, 1870–1950* (Chicago and London: University of Chicago Press, 2011), 171.

96 V.I. Khasnulin, ‘Vyi͡avlenie spet͡sificheskikh osobennosteĭ vozniknovenii͡a i techenii͡a ostrykh i khronicheskikh infekt͡sionnykh zabolevaniĭ v sisteme organov dykhanii͡a u korennogo i prishlogo naselenii͡a Kraĭnego Severa … ’, 14 May 1979, NCA, R-56/3/5; V.N. Nikolaev, et al. Vlii͡anie geofizicheskikh i meteorologicheskikh faktorov vneshneĭ sredy na organizm cheloveka v uslovii͡akh Zapadnoĭ Sibiri (programma Globėks-80), 1980, NCA, R-56/3/6.

97 Kaznacheev; Vlail’ P. Kaznacheev and Sergeĭ V. Kaznacheev, ‘Problemy adptat͡sii i konstitut͡sii cheloveka na Kraĭne Severe’, in *Klinicheskie aspekty poli͡arnoĭ medit͡siny*, ed. Vlail’ P. Kaznacheev (Moscow: Medit͡sina, 1986), 10–15 and other works.

98 Kaznacheev, *Sovremennye aspekty adaptat͡sii*, 145.

99 While building on the legacy of Grigoriy Danishevskiy, Vlail’ Kaznacheev took acclimatization studies in new directions. He re-designated Grigoriy Danishevskiy’s disadaptational meteoneuroses as ‘polar tension syndrome’ (*sindrom poli͡arnogo napri͡azhenii͡a*). By this term, Vlail’ Kaznacheev referred to a combination of geophysical, climatic, corporeal, and psychophysiological factors that affected migrants from non-Arctic regions.

100 Similar ideas were proposed in the 1930s, but they did not receive much enthusiasm from Soviet citizens at that time. See A. Bliumenfelʹd, ‘Zapoli͡arnyĭ kurort (lechebnai͡a arkticheskai͡a problema)’, *Vestnik znanii͡a*, no. 1 (1934): 37–43. This approach was repeated in the 1960s and was considered to be one of the most promising methods for acclimatizing settlers in the North. See Arnol’di, *Akklimatizat͡sii͡a cheloveka na severe i i͡uge*.

101 In the following publications, he openly acknowledges the impossibility of applying Indigenous knowledge to the development of the Siberian acclimatization project, Kaznacheev, *Sovremennye aspekty adaptat͡sii*, 26; Vlail’ P. Kaznacheev and Vladimir M. Strigin, *Problema adaptat͡sii cheloveka. Nekotorye itogi i perspektivy issledovaniĭ* (Novosibirsk: Sovetskai͡a Sibir’, 1978), 26.

102 See Iosif V. Davydov, *Triumf i tragedii͡a sovetskoĭ kosmonavtiki. Glazami ispytateli͡a* (Moscow: Globus, 2000).

103 See Vasiliy V. Boriskin, *Zhiznʹ cheloveka v Arktike i Antarktike* (Leningrad: Medit͡sina, 1973); Aleksandr L. Matusov, *Uslovii͡a zhizni i sostoi͡anie zdorovʹi͡a uchastnikov poli͡arnykh ėkspedit͡siĭ* (Leningrad: Gidrometeoizdat, 1979).

104 Correspondence between Vlail’ P. Kaznacheev and Yuri I. Borodin, 1986, GANO R-2063/1/858.

105 The last experiment Vlail’ Kaznacheev conducted was with a pseudo-scientific device also known as the Kozyrev mirrors. See: *Prostranstvo Kozyreva*, Documentary, 2001, https://youtu.be/l4aL--OG880. interestingly, the history of those experiments were known even to the Central Intelligence Agency (or CIA): **CIA** 24090-91JUL22/17.06.01/A10. See also Lauren Aratani, ‘CIA File on Russian ESP Experiments Released – but You Knew That, Didn’t You?’, *The Guardian*, January 27, 2021, sec. US news, https://www.theguardian.com/us-news/2021/jan/27/russian-esp-experiments-cia-memo.

106 See some rare publications of modern scholars in Russia: Yuri G. Solonin and Evgeniy R. Boyko, ‘Mediko-fiziologicheskie aspekty zhiznedei͡atelʹnosti v Arktike’, *Arktika: ėkologii͡a i ėkonomika* 1, no. 17 (2015): 70–5.

107 See Alla Bolotova and Florian Stammler, ‘How the North Became Home: Attachment to Place among Industrial Migrants in Murmansk Region’, in *Migration in the Circumpolar North: New Concepts and Patterns*, eds. Chris Southcott and Lee Huskey (Edmonton, Alberta: Canadian Circumpolar Institute Press and University of Alberta, 2010), 193–220.

108 Thompson, *Settlers on the Edge*.

109 Field ethnographers document intricate models of economic exchanges between shift workers and Indigenous communities: Elena Liarskaya, ‘“Where Do You Get Fish?”: Practices of Individual Supplies in Yamal as an Indicator of Social Processes’, *Sibirica* 16, no. 3 (December 1, 2017): 124–49, https://doi.org/10.3167/sib.2017.160306; Dmitry V. Arzyutov, ‘Oleni i/ili benzin: ėsse ob obmenakh v severo-i͡amal’skoĭ tundre’, in *Sot͡sial’nye otnoshenii͡a v istoriko-kul’turnom landshafte Sibiri*, ed. Vladimir N. Davydov (Saint Petersburg: MAĖ RAN, 2017), 314–48.

110 Rouillard, ‘Foreign Bodies in the Russian North’.

